# Supplementary material for: Mechanisms governing the pioneering and redistribution capabilities of the non-classical pioneer PU.1
Source: Nat Commun. 2020 Jan 21;11:402. doi: 10.1038/s41467-019-13960-2 (PMC6972792; doi:10.1038/s41467-019-13960-2)
Supplement: Supplementary file 7 — Source data [file 41467_2019_13960_MOESM7_ESM.zip › Source_Data/FigureS2/FigureS2B_MotifScanOutput/KmeansCluster6/homerResults/motif20.similar.html]

motif20

## Information for motif20

C
G
T
A
C
T
A
G
C
T
A
G
C
T
A
G
C
T
A
G
C
T
A
G
C
G
T
A
G
T
C
A
T
C
G
A
C
G
A
T
G
T
A
C
G
A
T
C
T
C
G
A
A
G
T
C
  
Reverse Opposite:  

A
C
T
G
A
G
C
T
C
T
A
G
C
A
T
G
G
C
T
A
A
G
C
T
A
C
G
T
A
C
G
T
A
G
T
C
A
G
T
C
A
G
T
C
G
A
T
C
A
G
T
C
G
C
A
T
  

|  |  |
| --- | --- |
| p-value: | 1e-51 |
| log p-value: | -1.176e+02 |
| Information Content per bp: | 1.743 |
| Number of Target Sequences with motif | 62.0 |
| Percentage of Target Sequences with motif | 1.88% |
| Number of Background Sequences with motif | 24.0 |
| Percentage of Background Sequences with motif | 0.06% |
| Average Position of motif in Targets | 104.2 +/- 64.6bp |
| Average Position of motif in Background | 86.2 +/- 40.7bp |
| Strand Bias (log2 ratio + to - strand density) | 0.2 |
| Multiplicity (# of sites on avg that occur together) | 1.00 |
| Motif File: | file (matrix) reverse opposite |

### Similar de novo motifs found

|  |  |  |  |  |  |  |  |
| --- | --- | --- | --- | --- | --- | --- | --- |
| Rank | Match Score | Redundant Motif | P-value | log P-value | % of Targets | % of Background | Motif file |
| 1 | 0.824 | A C T G C T A G A G C T T C G A C T A G C G T A A G C T A C G T C G A T A T G C G A T C G T A C G A T C | 1e-43 | -101.123134 | 1.91% | 0.10% | motif file (matrix) |
| 2 | 0.753 | A C T G C T A G C T A G A C T G C T A G C G T A C G T A G T C A G A T C A G T C T C A G T A G C A G T C | 1e-18 | -43.264740 | 1.12% | 0.12% | motif file (matrix) |
